# Green Tea Consumption and Risk of Pancreatic Cancer: A Meta-analysis

**DOI:** 10.3390/nu6114640

**Published:** 2014-10-28

**Authors:** Jin-Long Zeng, Zhi-Hua Li, Zhi-Chao Wang, Hai-Liang Zhang

**Affiliations:** 1 Department of Oncology, Zengcheng People’s Hospital, Boji Hospital of Sun Yat-sen University, Xingning, 511300, China; E-Mails: zengjinlonger@163.com (J.-L.Z.); wangzc1978@163.com (Z.-C.W.); zhanghailiang678@126.com (H.-L.Z.); 2 Department of Oncology, Sun Yat-sen Memorial Hospital, Sun Yat-sen University, Guangzhou, 510000, China

**Keywords:** green tea, pancreatic cancer, meta-analysis

## Abstract

Emerging laboratory and animal studies indicate that green tea inhibits development and progression of pancreatic cancer, but evidence from epidemiologic studies appears inconsistent and inconclusive. A meta-analysis summarizing published case-control and cohort studies was performed to evaluate the association of green tea consumption with risk of pancreatic cancer. Pertinent studies were identified by a search of PubMed and EMBASE up to April 2014. A random-effects model was assigned to compute summary risk estimates. A total of three case-control studies and five prospective studies were included, comprising 2317 incident cases and 288209 subjects. Of them, three studies were from China and the reminders were conducted in Japan. Overall, neither high *vs.* low green consumption (odds ratio (OR) = 0.99, 95% confidence interval [CI] = 0.78–1.25), nor an increase in green tea consumption of two cups/day (OR = 0.95, 95% CI = 0.85–1.06) was associated with risk of pancreatic cancer. The null association persisted when the analysis was stratified by sex or restricted to non-smokers. In the stratification by study location, the summary OR for the studies from China and for those from Japan was 0.77 (95% CI = 0.60–0.99) and 1.21 (95% CI = 0.94–1.54), respectively (*P* for differences = 0.04). Cumulative epidemiologic evidence suggests that green tea consumption is not associated with pancreatic cancer.

## 1. Introduction

Pancreatic cancer, which is currently the fourth leading cause of cancer death in men and women, is an aggressive malignancy characterized by poor prognosis and patient survival, with a 5-year survival rate of less than 5% [1]. Despite advances in surgery and radiation oncology, no significant improvement of the overall survival was achieved [1]. As there is no effective screening modality, primary prevention appears the most important way to reduce pancreatic cancer morbidity and mortality. However, apart from smoking and family history, little has be established about its etiology [1]. 

Green tea is one of the most popular beverages in Asia, particularly in China and Japan. Over the past 2 decades, potential chemopreventive effects of green tea constituents on cancer development have drawn substantial attention because of a large body of evidence from laboratory and animal studies continuously demonstrating a favorable effect of green tea on cancers of different sites, including pancreatic cancer [2,3,4,5,6]. Nevertheless, data from epidemiologic studies are less certain. The 2007 report from the World Cancer research Fund and American Institute for Cancer research concluded that the evidence that supports the benefits of green tea on cancer risk was “limited to suggestive” [7]. More recently, a Cochrane review on the prevention of cancer by green tea judges intake of three to five cups of green tea per day (up to 1200 mL/day, providing a minimum of 250 mg/day catechins) to be desirable [8]. However, limited epidemiologic evidence exists for the cancer of pancreas at the time of the review. To date, numerous case-control and cohort studies [9,10,11,12,13,14,15,16] have been performed to elucidate the role of green tea consumption with respect to its effect on pancreatic cancer development, with inconsistency remains. To further clarify this subject, a meta-analysis of observational studies was carried out. 

## 2. Experimental Section

### 2.1. Literature Search and Selection

All case-control and cohort studies published in English-language journals from 1966 to April 2014, reporting the association between green consumption and risk of pancreatic cancer were identified by searching PubMed and EMBASE using the search terms “green tea”, in combination with “pancreatic” or “pancreas”. The citation sections of recovered articles were also reviewed to identify additional studies. To be included, studies also had to report relative risks (RRs) or odds ratio (OR) with 95% confidence intervals (CIs) of pancreatic cancer associated with green tea consumption. 

### 2.2. Data Extraction 

For each study, the following characteristics were collected: author, publication year, country of origin, study design, sex of subjects, sample size, method for exposure measurement, the RRs or ORs and 95% CIs that reflected the greatest degree of control for potential confounders, and variables accounted for in the analysis. Study selection and data were carried out independently by two authors.

### 2.3. Statistical Analysis 

OR with 95% CI is the common measure of the association in this meta-analysis, and RR in the cohort studies were considered approximately as ORs. The results that were reported by sex separately were combined with a fixed-effects model, and the combined results were used in the meta-analysis. The random-effects model accounting for both within- and between-study variation was assigned to compute the summary risk estimates [17]. Subgroup analysis stratified by study design, number of cases, geographic area, sex and smoking status were also carried out. 

A dose-response analysis according to the method proposed by Greenland and Longnecker [18] and Orsini *et al*. [19] was also performed. For the studies [9,11] that assessed green tea consumption in terms of gram (g) of tea leaves, we rescaled tea intake to the number of cups per day by assuming 2.5 g tea leaves as approximately equivalent to one cup. For every study, the median or mean level of green tea for each category was assigned to each corresponding risk estimate. When the median or mean level was not reported, we assigned to each class the dose corresponding to the midpoint of upper and lower boundaries. When the highest or lowest category was open-ended, we assumed the width of the interval to be the same as in the closest category. 

Statistical heterogeneity was assessed using Q and *I*^2^ statistics [20]. For the Q statistic, a *P*-value of less than 0.1 was considered statistically significant heterogeneity; and for the *I*^2^ statistic, we set the following cut-off points: <25% (low heterogeneity), 25%–50% (moderate heterogeneity) and >75% (severe heterogeneity). Potential publication bias was evaluated using Egger’s test and Begg’s funnel plot [21]. All statistical analyses were performed using STATA software, version 11.0 (StataCorp, College Station, TX, USA). 

## 3. Results 

### 3.1. Study Characteristics

**Figure 1:**
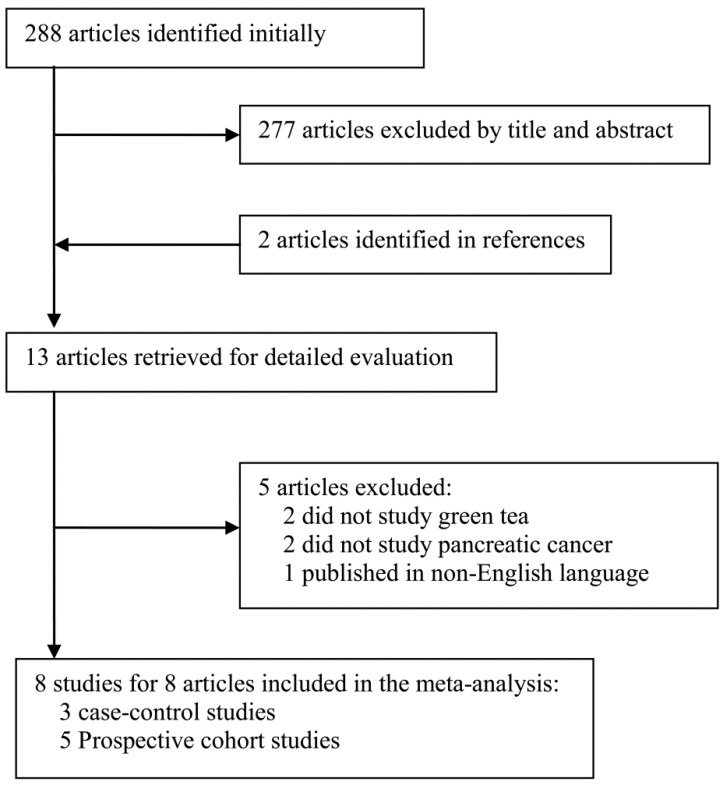
Selection of studies for inclusion in the meta- analysis.

The search strategy yielded 288 citations (Figure 1), of which 13 potentially relevant articles were retrieved and assessed in more detail. Finally, a total of 8 observational studies [9,10,11,12,13,14,15,16], including three case-control studies and five prospective studies that met our eligibility criteria were included. Agreement between reviewers was excellent (Kappa statistic = 0.79). The 8 studies, whose total size comprised 2317 incident cases and 288,209 subjects, were published between 1992 and 2012. Three studies were from china and the remaining five studies were from Japan. Two out of the three case-control studies were population based studies, and all but one prospective cohort study were followed up for >10 years. The main characteristics of the included studies are summarized in Table 1. 

The reported findings on the association of green tea consumption and risk of pancreatic cancer among individual studies varied substantially. An overall significant/non-significant inverse association were reported in three studies [9,11,14] (OR ranged from 0.53 to 0.79), but a positive association was also found in the hospital based case-control study [10] from Japan (OR = 1.94). Luo *et al*. [13] and Nakamura *et al*. [15] found a non-significant positive association in men (OR = 1.4 and 1.77, respectively), whereas a null (OR = 1.0) or a non-significant inverse association (OR = 0.59) in women, which contradicted the results reported by Lin *et al*. [12] (OR was 0.95 in men and 1.54 in women). Finally, the Shanghai Women’s Health Study (SWHS) [16] found that regular green consumption (defined as ≥3 times per week for >6 month) was not associated with risk of pancreatic cancer in non-smoking andnon-alcohol drinking women. 

### 3.2. Meta-analysis

**Figure 2:**
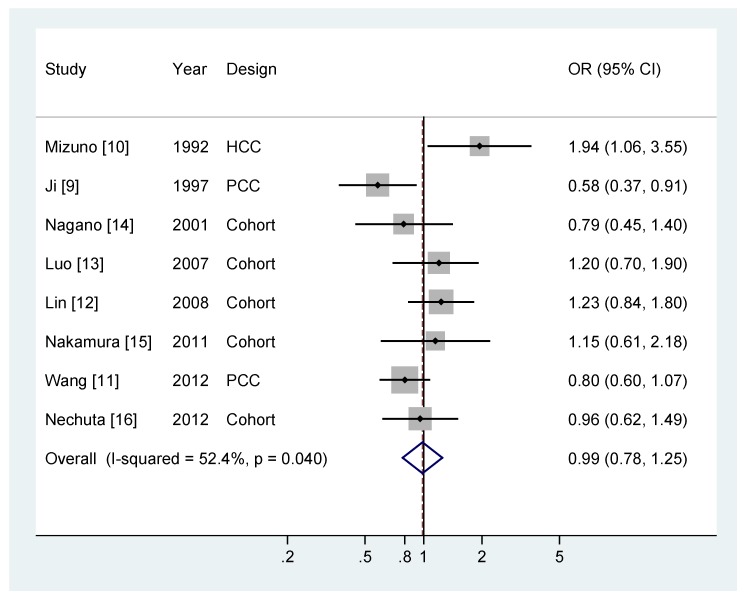
The odds ratio of pancreatic cancer for the highest *versus* lowest categories of green tea consumption for each study and all studies combined [9,10,11,12,13,14,15,16].

Figure 2 shows OR and 95% CI of pancreatic cancer for the highest compared with lowest categories of green tea consumption. The summary OR was 0.99 (95% CI = 0.78–0.25), suggesting that green tea consumption was not associated with risk of pancreatic cancer. There was some evidence of heterogeneity (*P* = 0.04, *I*^2^ = 52.4%). Results of Egger’s test indicated no evidence of publication bias (*P* = 0.31), which could be supported by a visual examination of Begg’s funnel plot (not shown). 

**Table 1:** Characteristics of published case-control and cohort studies on green tea consumption and pancreatic cancer.

Study	Country	Design	Sex	Cases/participants	Green tea consumption (highest *vs*. lowest)	OR/RR (95% CI)	Exposure assessment	Variables controlled for
Mizuno, 1992 [10]	Japan	HCC	M/F	127/254	≥5 *vs.* <5 cups/day	1.94 (1.06–3.55)	Interview	Age and sex.
Ji, 1997 [9]	China	PCC	M/F	451/2003	≥300 *vs.* 0 g/month (M) ≥200 *vs*. 0 g/month (F)	0.63 (0.34–1.17) (M) 0.53 (0.29–1.09) (F)	Interview	Age, income, education and smoking.
Nagano, 2001 [14]	Japan	Cohort	M/F	122/3854	≥5 *vs*. 0–1 cups/day	0.79 (0.45–1.40)	Self-report	Age, sex, BMI, radiation dose, drinking history, education and calendar time.
Luo, 2007 [13]	Japan	Cohort	M/F	233/102137	≥ 5 *vs*. 0 cups/day	1.20 (0.70–1.90)	Self-report	Age, sex, BMI, physical activity, smoking, history of diabetes or cholelithiasis, study area, and intakes of coffee and alcohol.
Lin, 2008 [12]	Japan	Cohort	M/F	292/77850	≥7 *vs*. <1 cups/day	1.23 (0.84–1.80)	Self-report	Age, sex, BMI, smoking, alcohol drinking, and history of diabetes or gallbladder diseases.
Nakamura, 2011 [15]	Japan	Cohort	M/F	52/30826	≥1 *vs*. 0 cups/day	1.77 (0.78–4.04) (M) 0.59 (0.21–1.61) (F)	Self-report	Age, BMI, smoking, and history of diabetes.
Wang, 2012 [11]	China	PCC	M/F	908/1975	≥250 *vs*. 0 g/month (M) ≥150 *vs*. 0 g/month (F)	0.91 (0.65–1.27) (M) 0.56 (0.32–0.98) (F)	Interview	Age, BMI, education, family history of cancer, smoking, history of type 2 diabetes, and additional adjustment for women included: menopausal status, oral contraceptives use, and menopausal hormone therapy.
Nechuta, 2012 [16]	China	Cohort	F	132/69310	Regular *vs*. Never regular.	0.96 (0.62–1.49)	Self-report	Age, BMI, marital status, education, occupation, exercise, intakes of fruit, vegetable and meat, history of diabetes, and family history of digestive system cancer.

BMI, body mass index; CI, confidence interval; F, female; HCC, hospital based case-control; M, men; OR, odds ratio; PCC, population based case-control; RR, relative risk.

Table 2 is a presentation of the results of subgroup analysis. The null association between green tea consumption and risk of pancreatic cancer was continuously observed, with an exception among Chinese population. An analysis of three studies from china suggested that subjects with higher green tea consumption had 33% lower risk of developing pancreatic cancer (OR = 0.77, 95% CI = 0.60–0.99). Conversely, there was a trend toward an increase in odds of pancreatic cancer for subjects with high consumption in Japan (OR = 1.21, 95% CI = 0.94–1.54). Meta-regression analysis indicated that study location may be a source of heterogeneity (*P* for interaction = 0.04).

**Table 2:** Stratified analysis on the association of green tea consumption (highest *versus* non/lowest) and risk of pancreatic cancer.

	*N*	OR (95% CI)	*P* for heterogeneity	*I*^2^ (%)	*P* for differences
All studies	8	0.99 (0.78–1.25)	0.04	52.4	
Design					
Cohort	5	1.07 (0.87–1.33)	0.72	0.0	0.52
Case-control	3	0.93 (0.53–1.63)	0.006	80.2	
Number of cases					
>200	4	0.90 (0.65–1.25)	0.04	63.2	0.44
<200	4	1.11 (0.78–1.60)	0.17	41.0	
Area					
China	3	**0.77 (0.60–0.99)**	0.23	21.7	**0.04**
Japan	5	1.21 (0.94–1.54)	0.34	12.0	
Sex					
Men	5	0.98 (0.74–1.30)	0.27	22.3	0.50
Women	6	0.84 (0.59–1.19)	0.08	49.9	Reference
Both sexes	4	1.22 (0.89–1.65)	0.21	33.6	
Smoking status					
Smokers	1	0.90 (0.45–1.80)	-	-	0.84
Non-smokers	3	1.01 (0.65–1.57)	0.13	50.7	

CI, confidence interval; *N*, number of included studies; OR, odds ratio.

Adjustment for cigarette smoking is important because smoking often highly correlated with tea consumption, especially among Asian population. Restricting the analysis to the studies that took into account smoking, the summary OR was 0.93 (95% CI = 0.74–1.18). Three studies [11,12,16] also evaluated the effect of green tea consumption among non-smokers, and the summary RR for non-smokers was 1.01 (95% CI = 0.65–1.57). 

There were six studies [9,11,12,13,14,15] that provided sufficient data for the dose-response analysis. The summary OR of pancreatic cancer for an increase in green tea consumption of two cups/day was 0.95 (95% CI = 0.85–1.06), and there was significant heterogeneity (*P* = 0.04, *I*^2^ = 56.9%).

## 4. Discussion 

To our knowledge, the present study is the first quantitative analysis of the association between green tea consumption and risk for pancreatic, based on published results from 8 observational studies involving more than 2300 cases. Overall, our findings suggest that green tea consumption is not associated with risk of pancreatic cancer. Interestingly, a 33% reduction in risk associated with high green tea consumption was observed in Chinese population. 

Tea polyphenols, especially (−)-epigallocatechin-3-gallate (EGCG), which is an active ingredient in green tea, have been shown to have antimutagenic, antigenotoxic, and anticarcinogenic properties [2]. It is suggested that oxidative stress resulting from an imbalance between pro- and antioxidants is an important mechanism involved in the development of cancer [22]. Since flavanols in green tea have strong antioxidant properties [6], consuming green tea may contribute to protection from oxidative stress, and thus contribute to inhibition of cancer development. For pancreatic cancer, components from green tea extracts including EGCG have been found to effectively inhibit growth and induces apoptosis in human pancreatic cancer cells [3,4,5,23] and suppress the process of pancreatic carcinogenesis in animal studies [24,25]. 

The differences between experimental and epidemiologic findings may be attributable to several reasons. On one hand, the amount of green tea consumed in human studies may not be large enough to reach an effective dose inhibiting pancreatic carcinogenesis because the doses of tea polyphenols used in some of experimental studies are much higher than those observed in human plasma and tissue after green tea consumption [26]. On the other hand, when placed in the context of the whole dietary pattern, green tea consumption may correlate with other lifestyle habits (unadjusted confounding factors) that influence the risk of pancreatic cancer, or interact with other components in food in unknown mechanisms [27]. In addition, a potential recall bias in case-control studies might have weakened the effects of factors relating to suppressing diseases, and measuring green tea with self-administrated food frequency questionnaires only once at baseline in cohort studies may have led to non-differential misclassification, which may also attenuate the true association towards the null. 

When stratifying the analysis by geographic area, we found a significant inverse association of green tea consumption with risk of pancreatic cancer among Chinese population, but a positive (non-significant) one among Japanese. Similar results for colorectal cancer have been reported in a recent meta-analysis [28]. Differences in tea preparation (generally, tea is processed by dry roasting in China and by using steam in Japan), which may result in different bioactivity of green tea consumed between countries [29], may be one of explanations for this disparity. Moreover, differences in lifestyle factors linked to green tea drinking may also be a possibility. Noteworthy is that the inverse association observed in Chinese population was mainly driven by two retrospective case-control studies [9,11] which are prone to biases, and therefore should be treated with caution. In addition, the inverse association may simply occur by chance given the multiple tests performed. 

Smoking has been suggested to be an important factor confounding the association of tea consumption and cancer risk because green tea drinkers, in particular male drinkers were more likely to be smokers [9,11]. Possible effect modifications by sexes have been reported for oesophageal cancer [30], stomach cancer [31] and colorectal cancer [32]. For pancreatic cancer, however, no such differences were found between men and women when stratifying the analysis by sex (*P* for differences = 0.50). A pooled analysis of three studies [11,12,16] of non-smokers also yielded a summary of 1.01 (95% CI = 0.65–1.57), which to some extent excluded the possibility of confounding by smoking. Nevertheless, the impacts of residual confounders associated with different behaviors between sexes still cannot be ruled out. 

The relatively low incidence of pancreatic cancer compared with other common cancers results in small number of incident cases in cohort studies. Therefore, individual studies have been underpowered to examine this relation across a broad range of green tea consumption. This meta-analysis quantitatively summarizing cumulative evidence from published cohort and case-control studies comprising more than 2300 cases largely enhanced the statistical power. However, except for possible influences of residual confounding and exposure misclassification mentioned above, several other limitations should also be acknowledged. First, the levels and the ranges of green tea consumption, as well as statistical adjustment in primary studies differed substantially, which may have contributed to heterogeneity amongst studies. Second, it is possible that cancer patients might have altered their recent tea drinking habits as a result of their pre-clinical illness, which may bias our findings. Three cohort studies [12,13,14] had repeated the analyses by omitting the data for the first 2 or 3 years of follow-up, and no substantial change in any result was found. Finally, potential publication bias also merits consideration since our study is based on published literature. Despite no evidence of such bias observed, the tests including small number of studies may be of limited power. 

## 5. Conclusions 

In summary, findings from the present meta-analysis indicate that green tea consumption is not associated with risk of pancreatic cancer. The results of lower risk associated with high consumption among Chinese population were mainly derived from case-control studies, and so warrant further confirmation. 
